# Intrauterine cytomegalovirus infection: a possible risk for cerebral palsy and related to its clinical features, neuroimaging findings: a retrospective study

**DOI:** 10.1186/s12887-020-02449-3

**Published:** 2020-12-08

**Authors:** H Xu, L Zhang, XY Xuan, M Zhu, J Tang, XK Zhao

**Affiliations:** Department of Rehabilitation, Children’s Hospital of Nanjing Medical University, Nanjing, China

**Keywords:** Cerebral palsy, Cytomegalovirus, Mental deficiency, Pediatric

## Abstract

**Background:**

Abundant clinical evidences indicate that the increased risk of cerebral palsy (CP) may be associated with the intrauterine exposure to maternal infection. Cytomegalovirus (CMV) is a common cause of CP. However, little is known about the relationship between the intrauterine exposure of the fetus to CMV infection and CP. This study aims to explore the relationships between intrauterine CMV infection and clinical symptoms, classification, intelligence development and brain neuroimaging findings in children with CP.

**Methods:**

In this study, 147 children with CP in recent 6 years were retrospectively analyzed (average age: 14.76 ± 3.07months; sex (M/F): 103/44). 148 children had CMV IgG and IgM positive sera identified by TORCH examination were selected as the control group (average age: 15.10 ± 3.21months; sex (M/F): 102/46), which also undergo the examination of CMV-DNA in urine. The age and sex of children in the control group were matched with those in the CP group. CMV-DNA in urine was detected by CMV fluorescence quantitative PCR, and *t*-test was performed to analyze the number of copies. For the CP group, standardized rehabilitation treatment was performed and the function of gross motor was evaluated by GMFM scale before and after treatment. The Gesell developmental scale (GDS) was used to assess the level of intellectual development. The classification of CP was conducted and the results of magnetic resonance imaging were analyzed. Finally, the correlations between the copy number of CMV-DNA and the clinical characteristics of children with CP were evaluated by the method of *Pearson* and *Spearman* correlation analysis.

**Results:**

The level of CMV infection was negatively correlated with the developmental quotient (DQ) of children with CP. Negative association was found between the level of CMV infection and the level of the gross motor development. The level of CMV infection was positively related with the occurrence probability of spastic quadriplegia. However, no associations were found between the abnormalities of brain tissue and the number of CMV copies. Moreover, CMV infection might add the difficulty of the rehabilitation treatment.

**Conclusions:**

CMV infection is a risk factor for the occurrence of CP in children. Pregnancy examination should be strengthened. Early detection and control of CMV infection may contribute to the rehabilitation of children with CP and reduce the disability and social burden.

## Background

Cerebral palsy (CP), a common and significant physical disability, is defined as a group of permanent disorders, which is caused by the non-progressive disturbances of the developing fetal or infant brain [1–4]. CP is mainly identified by a range of motor problems including the movement and posture disorders, which cause the limitations in mobility [5–7]. Moreover, the accompanying neurological and developmental disorders of CP including disturbances of cognition, behaviour, communication, sensation, perception and epilepsy, secondary musculoskeletal problems, which may also have a greater effect on the quality life of children with CP [8, 9].

Maternal infections during the pregnancy is a common cause of CP [10–12]. Previous studies demonstrated that the intrauterine exposure to infection, particularly cytomegalovirus (CMV), might be responsible for most cases of post-neonatally acquired CP [13–15]. The maternal infection-related factors are considered as risk factors for the subtype spastic hemiplegia CP, but not for the spastic diplegia, tetraplegia, or dyskinetic CP [16]. In addition, other previous studies showed that the maternal infections were involved in causing spastic CP while the neonatal infections might be associated with the bilateral spastic and dyskinetic CP [13, 17]. The inflammatory in the fetus is known as fetal inflammatory response syndrome, which can be activated by the maternal infection and is mediated by different cytokines [18]. The fetal inflammatory response syndrome can induce neonatal white matter injury, which is a cause of fetal or neonatal injury that leads to motor impairment of CP in preterm-born children [19].

Substantial evidences demonstrate that both direct infection and infection-triggered damage can elicit the brain inflammation and innate immune response, which are closely associated with both acute and chronic brain disease in the newborn [20, 21]. Therefore, exposure to neonatal infection can lead to an infection-related inflammatory response, which is a great contributor to the injury of the brain [22, 23]. In addition, previous studies showed that the lesions of white matter in the neonatal brain were the most important identifiable risk factor for CP [24, 25]. CP is often caused by the damage of the developing brain [26, 27]. The intrauterine infections can lead to the release of inflammatory cytokines, which may contribute to the brain white matter lesions and CP [28–30]. The damage of white matter in the brain can be also caused by the cytokine in the amniotic fluid, which is associated with CP for children born at term [31, 32]. Moreover, previous neuroimaging studies suggested that the infection was associated with the cerebrovascular ischemia, which might lead to the white matter lesion of the cortical or subcortical brain regions, which was also an important cause of motor impairment of CP [4, 33]. An increased risk of brain abnormalities was found in the infants with CMV, which resulted in severe damage to the developing brain of children with CP [22, 34]. However, little is known about the relationship between the intrauterine cytomegalovirus infection and the clinical features, neuroimaging findings of CP. Therefore, more informations about the associations between the causes of CP and the infection in the perinatal period are needed.

The aims of our study were to determine whether CMV was a possible risk for cerebral palsy and whether CMV was related to the clinical features, neuroimaging findings of children with CP. Therefore, the clinical characteristics and CMV-DNA in urine of were detected in the group of children with CP and the control group. Differencs between groups and the relationships between CMV and the clinical features, neuroimaging findings were evaluated.

## Methods

### Participants

This study retrospectively analyzed the children with CP diagnosed for the first time in the rehabilitation department of our hospital from January 2011 to December 2016. Gender- and age-matched children with CMV IgG and IgM positive sera identified by TORCH examination were collected as the control group. In addition, CMV-DNA in urine of these children were also collected. This study was approved by the ethical commission of Children’s Hospital of Nanjing Medical University (Program number (81,501,946): Version 1). Additionally, consents were obtained from parents or guardians on behalf of participants.

The diagnostic criterias of CP consisted of four necessary conditions and two reference conditions [35]. Necessary conditions: (1) central dyskinesia appeared uninterruptedly; (2) abnormal development of movement and posture; (3) abnormal development of reflex; (4) abnormal muscle tension and muscle strength. Reference conditions: 1) pathological basis leading to CP; (2) brain imaging evidences (magnetic resonance imaging (MRI)).

Inclusion criterias for participants were as follows: (1) children who met the diagnostic and typing criteria of CP and did not receive any treatment before the diagnosis; (2) CMV-IgG and IgM in the serum of mother was positive identified by TORCH test during pregnancy, CMV-IgG and IgM in the serum of children was positive identified by TORCH test, and the urinary CMV-DNA data was also collected; (3) CMV infection was not treated with antiviral drugs; (4) all patients received standardized rehabilitation treatments after the diagnosis, such as neurodevelopmental stimulation, muscle spasm/muscle excitation, cerebral circulation, meridian leveling, magnetic therapy, brain ultrasound, biofeedback, muscle strength training and neurotrophic drugs in the rehabilitation department of our hospital; (5) MRI data were collected at the average age of 14.76 ± 3.07 months for children with CP and at the average age of 15.10 ± 3.21 months for the control children.

Exclusion criterias for participants were as follows: (1) children with CP caused by neonatal hypoxic ischemic encephalopathy (HIE), premature birth, nuclear jaundice and chromosomal abnormalities among high-risk factors; (2) CMV infection had been treated with antiviral therapy; (3) standardized treatment had not been completed due to special conditions such as epilepsy.

**Fig. 1:**
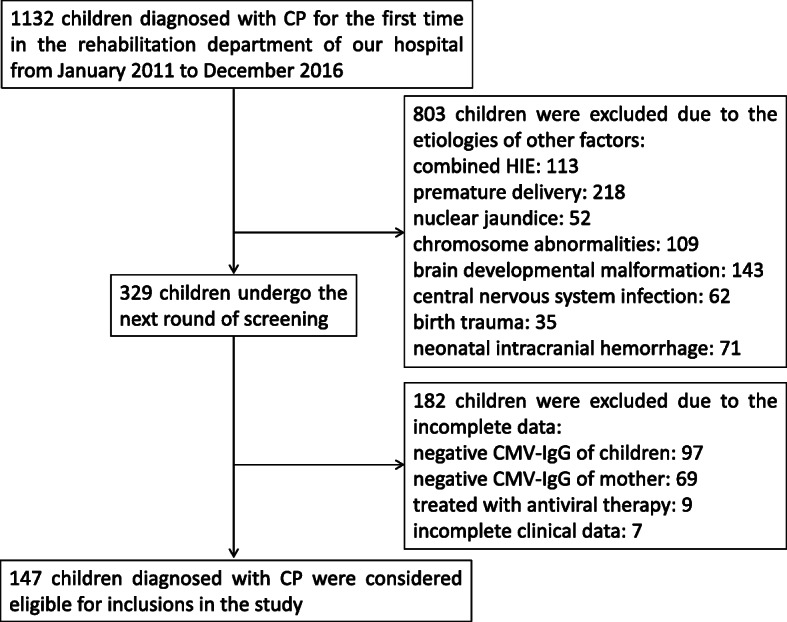
Flow diagram of data collection

Initially, 1132 children diagnosed with CP in the rehabilitation department of our hospital from January 2011 to December 2016 were collected. During the first round of screening, 803 children were excluded due to the etiologies of other factors (combined HIE: 113, premature delivery: 218, nuclear jaundice: 52, chromosome abnormalities: 109, brain developmental malformation: 143, central nervous system infection: 62, birth trauma: 35, neonatal intracranial hemorrhage: 71). After the second round of screening, 182 children were excluded due to the incomplete data (negative CMV-IgG and IgM of children: 97, negative CMV-IgG and IgM of mother: 69, treated with antiviral therapy: 9, incomplete clinical data: 7). Finally, 147 children diagnosed with CP were considered eligible for inclusions in the study. The flowchart of the data collection was presented in Fig. 1.

### Detection of CMV-DNA in urine

Firstly, 1 ml mid-stream clean-catch urinary of all participates were collected in the morning. Then the urinary sediment was collected for DNA extraction after 12,000 r/min centrifugation for 5 min.

CMV-DNA was evaluated by CMV fluorescence quantitative PCR (FQ-PCR). The kit was a product produced by the banner of the Daan gene. The fluocycle real-time quantitative PCR (Real-Time System) was produced by the company of BIO-RAD in USA. The detection of CMV-DNA in urine was carried strictly according to the instructions. The standard curve was set by using the software of the company of BIO-RAD in US. The number of copies of samples were computed by setting standard curves with different concentrations of reference substances.

### Standardized rehabilitation therapy

All the children in the CP group received standardized rehabilitation treatment, including neurodevelopmental stimulation, muscle spasm/muscle excitation, cerebral circulation, meridian leveling, magnetic therapy, brain ultrasound, biofeedback, muscle strength training and neurotrophic drugs. The standardized treatment was composed of three courses of rehabilitation, and each course lasted for 21 days with an interval of one month.

### Therapeutic evaluation method

#### Gross motor function measure scale (GMFM)

The scale of GMFM [36] was aimed to find out the gross motor state of CP children, the changes of motor function caused by the disease and intervention. GMFM has strong validity and reliability, which can quantitatively reflect the changed trend of gross motor function in CP children. GMFM can be divided into five subscales with a total of 88 items (improved scale included 66 items). The five subscales are lying and turning, sitting, climbing and kneeling, standing, walking and jumping. The total score of the first subscale is 51. The total score of the second subscale is 60. The total score of the climbing and kneeling subscale is 42. The total score of the standing subscale is 39. The total score of the last subscale is 72. The results of GMFM is composed of the following parts: (1) original score: original score of all subscales; (2) total percentage: the sum of the percentage of the original score of all subscales/5; (3) monthly percentage: (total percentage of this time-previous total percentage)/number of interval months; (4) relative percentage of month: (current monthly percentage/previous total percentage)*100%. The function of gross motor before and after treatment was evaluated by GMFM scale.

#### Gesell developmental scale (GDS)

The developmental quotient (DQ) was evaluated by the Gesell developmental scale (GDS) (1986) [37]. All CP subjects were evaluated by psychological evaluators who had experienced professional training and long-term practical experience. All testing items were carried out step by step in a specially designated psychological assessment environment. The normal range: ≥86; the borderline state: 76 ~ 85; the mild mental retardation: 55 ~ 75; moderate mental retardation: 40 ~ 54; severe mental retardation: ≤39.

### Classification of CP

According to the types of dyskinesia and the location of paralysis, there are six types: spastic quadriplegia, spastic diplegia, spastic hemiplegia, dyskinesia, ataxic and mixed. The above types of spasticity are classified according to ICD-10 and the latest international reports on CP.

### Statistical analysis

Due to the great differences of CMV copy number, the logarithmic transformation was conducted. We performed *t*-test and variance analysis to compare the differences of CMV-DNA copy number, clinical typing and DQ of CP. Moreover, the associations between CMV-DNA copy number and the clinical characteristics of children with CP were assessed by the method of *Pearson* and *Spearman* correlation. The significance level was set at *P* < 0.05.

## Results

### Demographic and clinical characteristics

The average age of CP group was 14.76 months (Male/female: 103/44). The average GDS score of CP was 73.29, and the average GMFM score was 62.01. The average age of control group was 15.10 months (male/female: 102/46). There were no significant group-differences of gender (*t* = 0.93, *P* = 0.35) and age (χ^2^ = 0.07, *P* = 0.79). A summary for the detailed demographic and clinical characteristics of all participants were presented in Table 1.

**Table 1:** Demographic and clinical characteristics of participates

Variables	CP group (*n* = 147)	Control group (*n* = 148)	*t*/χ^2^ value	*P* value
**Average age (months)**	14.76 ± 3.07	15.10 ± 3.21	0.93	0.35^a^
**Sex (M/F)**	103/44	102/46	0.07	0.79^b^
**Average GDS score**	73.29 ± 12.73	-	-	-
**Average GMFM score**	62.01 ± 16.99	-	-	-
**Classification of CP**				
Spastic quadriplegia	14(9.6%)	-	-	-
Spastic diplegia	64(43.5%)	-	-	-
Spastic hemiplegia	38(25.9%)	-	-	-
Mixed type	20(13.6%)	-	-	-
Dyskinetic cerebral palsy	11(7.5%)	-	-	-
Ataxia type	2(1.4%)	-	-	-
**MRI findings**				
White matter damage	34(22.45%)	-	-	-
Brain dysplasia	24(15.65%)	-	-	-
Hydrocephalus	6(4.08%)	-	-	-
Encephalomalacia	9(7.48%)	-	-	-
Callosal dysplasia	23(15.6%)	-	-	-
Myelin hypoplasia	15(10.20%)	-	-	-
Normal structure	24(24.49%)	-	-	-

*CP* cerebral palsy, *GMFM* gross motor function measure scale, *GDS* Gesell developmental scale, *MRI* magnetic resonance imaging. ^a^*P* value was based on the independent sample *t* test. ^b^*P* value was based on the *Pearson* chi-square test. *P* value was significant at < 0.05

In the CP group, there were 14 cases (9.6%) of spastic quadriplegia, 64 cases (43.5%) of spastic diplegia, 38 cases (25.9%) of spastic hemiplegia, 20 cases (13.6%) of mixed type and 11 cases (7.5%) of dyskinetic cerebral palsy. For MRI findings, there were 34 cases (22.45%) of white matter damage, 24 cases (15.65%) of brain dysplasia, 6 cases (4.08%) of hydrocephalus, 9 cases (7.48%) of encephalomalacia, 23 cases (15.6%) of callosal dysplasia, 15 cases (10.20%) of myelin hypoplasia and 36 cases (24.49%) of normal structure.

### Differences of CMV copy number between groups

**Fig. 2:**
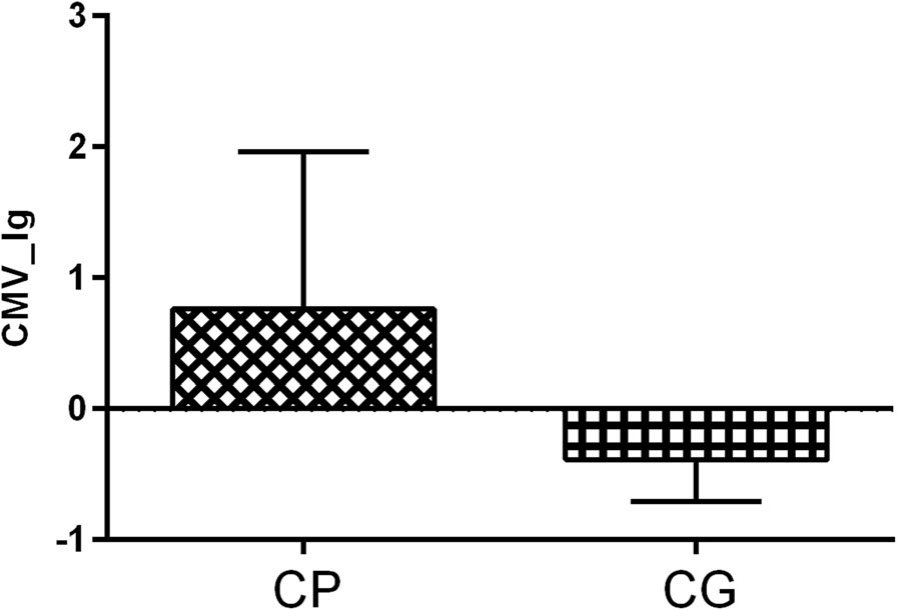
Differences of CMV copy number between cerebral palsy group and control group

The logarithmic transformation was conducted for the copy number of CMV. Compared with the control group (-0.39 ± 0.32), the CP group showed increased copy number of CMV (0.76 ± 1.20) (*t* = 11.21; *P* = 0.00) (Fig. 2).

### Differences of CMV copy number among different types of CP

**Fig. 3:**
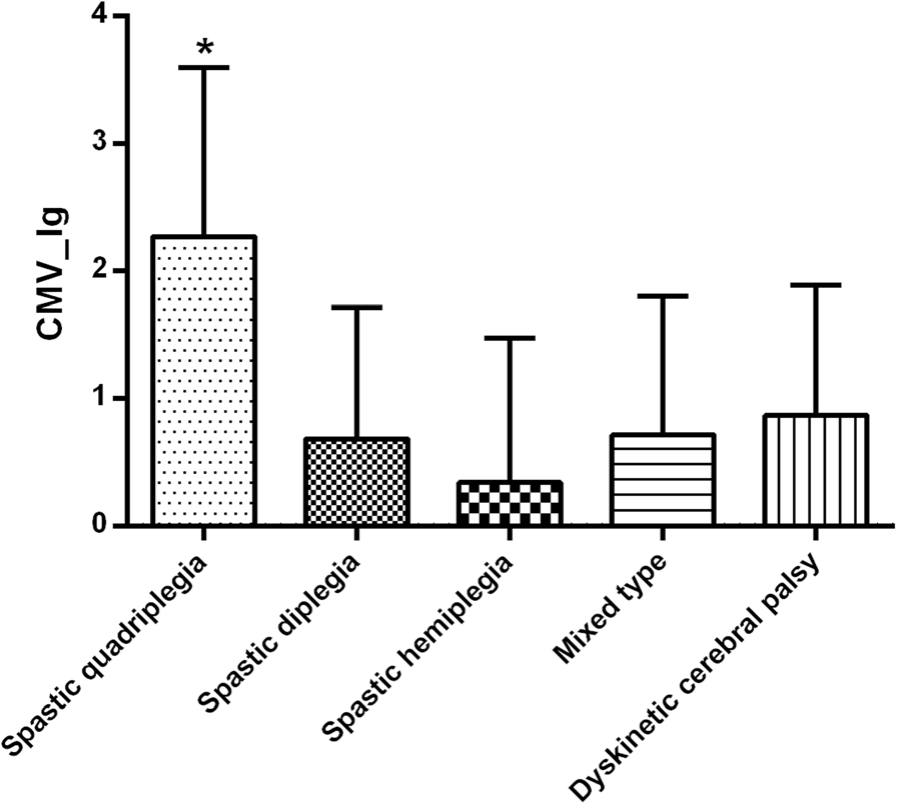
Differences of CMV copy number among different types of CP

The results of ANOVA analysis showed that there was significant difference of CMV copy number among different types of CP (*F* = 8.12; *P* = 0.000006). Children with spastic quadriplegia CP had higher rate of intrauterine CMV infection than other types of CP (*P* = 0.000003) (Fig. 3). However, there was no difference of intrauterine CMV infection rate among other types of CP (Fig. 3).

### Differences of CMV copy number among CP with different MRI findings

**Fig. 4:**
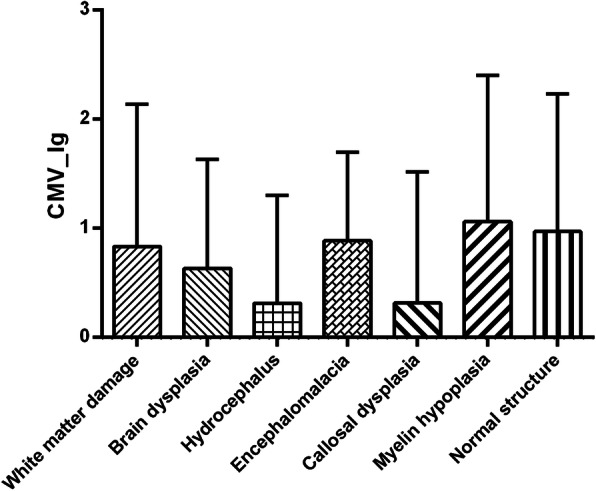
Differences of CMV copy number among CP with different MRI findings

ANOVA analysis demonstrated that there was no significant difference of CMV copy number among CP with different MRI findings (*F* = 1.10; *P* = 0.39) (Fig. 4).

### Relationships between CMV infection and clinical symptoms

**Fig. 5:**
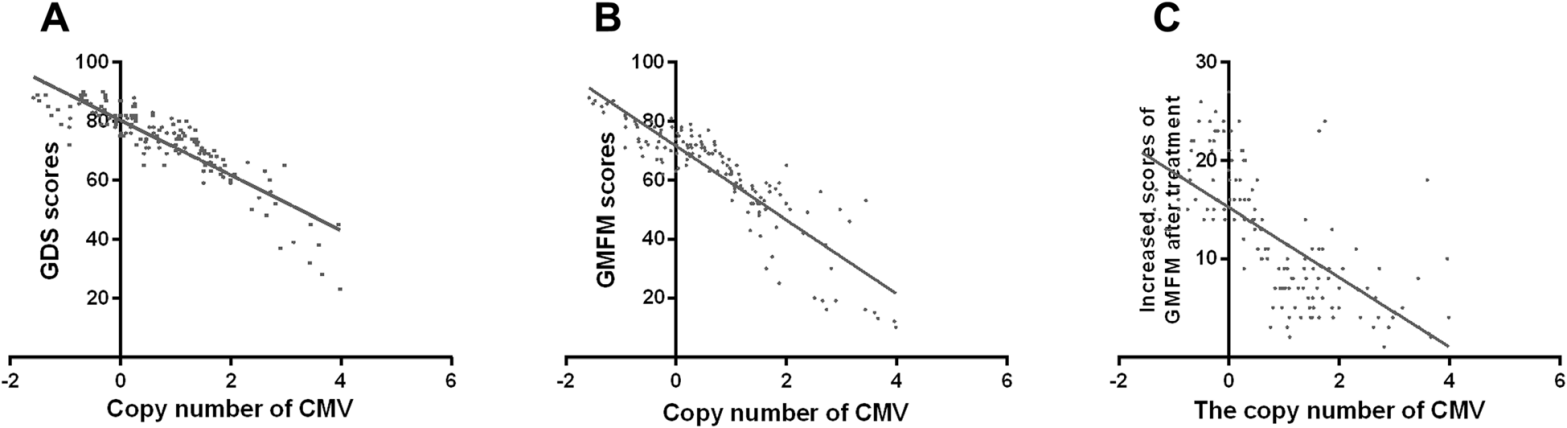
Relationships between CMV infection and clinical symptoms

Negative relationship was found between the copy number of CMV and the scores of GDS in the CP group (*r*=-0.88; *P* = 0.00) (Fig. 5A). In addition, CMV copy number of the CP group showed negative relationship with the GMFM scores of the CP group(*r*=-0.89; *P* = 0.00) (Fig. 5B).

Moreover, all the children in the CP group received standardized rehabilitation treatment for 6 months. All children with CP were evaluated by the scale of GMFM after the standardized rehabilitation treatment. The differences of GMFM scores before and after the treatment were considered as the curative effect index of CP children. The copy number of CMV was found to be negatively associated with the increased scores of GMFM after the treatment (*r*=-0.65; *P* = 0.00) (Fig. 5C).

## Discussion

CP is a common childhood physical disability of motor development, which is considered to be associated with a number of risk factors, including intrauterine CMV infection [35]. However, the relationships between CP and its clinical symptoms remain unknown. Our findings demonstrated that intrauterine CMV infection was a risk factor for CP and spastic quadriplegia CP patients who had higher rate of intrauterine CMV infection. The results also provided new knowledges about the associations between intrauterine CMV infection and the severity of clinical symptoms, the curative effect of standardized rehabilitation treatment of CP.

Multiple risk factors are associated with the development of CP [38]. CMV infection occurs in a large number of newborn infants [39]. CMV infection can cause damage to the developing brain and the infants with CMV infection are at increased risk of permanent neurodevelopmental disabilities including CP [40]. Intrauterine CMV infection can cause neurodevelopmental impairments and it is a predictor of poor cognitive outcome [41]. Intrauterine CMV infection can also reflecte the negative effect of intrauterine CMV infection on early neurogenesis [42]. Previous studies showed that infection in the perinatal period was involved in causing unilateral spastic CP, while neonatal infection was associated with bilateral spastic CP [17, 43]. The results of our study showed that CP patients had increased copy number of CMV when compared with the control group. Therefore, intrauterine CMV infection may increase the risk of CP. Infants with intrauterine CMV infection are more likely to have CP than children who do not have infection.

Infection is one of the most cases of post-neonatally acquired CP in developed countries [44]. Intrauterine exposure to infection in the latter stage of pregnancy is a strong risk factor for CP, especially CMV [45]. Moreover, neonatal infection increases the risk of spastic diplegia and the more severe form tetraplegia [17]. Previous study fund that infection was a risk factor for cerebrovascular ischemia [46]. The mechanism underlying this type of cerebrovascular ischemia may be the inflammatory, which stimulates coagulation and exacerbates the prothrombotic state during pregnancy and stimulate coagulation [47]. Maternal infection-related factors may activate an inflammatory response and infections should be taken seriously and treated accurately [48].

GMFM is a validated tool to measure gross motor function and evaluate the changes in the gross motor function of CP children [36]. In this study, spastic quadriplegia CP patients had higher rate of intrauterine CMV infection and negative associations were found between CMV and the of clinical symptoms of CP. In addition, intrauterine CMV infection was also negatively related with the curative effect of rehabilitation treatment in the CP group. Individuals with CP experience a range of impairments, especially variation in gross motor function [49]. The major characteristic of CP is the impaired development of gross motor function, which is related to the cognitive, auditory, visual defects of CP and is considered an indicator of prognosis [50]. Rehabilitation treatment has been used to improve the motor, cognitive and other function of CP patients [51]. In this study, prominent improvement was observed in CP children, which was reflected by the changes of GMFM score.

For the results of MRI findings, there were 24 cases (15.65%) of brain dysplasia, 6 cases (4.08%) of hydrocephalus. Previous neuroimging study showed that the dominant changes identified on MRI scans in children with CP were periventricular leukomalacia (42%) and posthemorrhagic hydrocephalus (21%) [52]. Another brain magnetic resonance imaging (MRI) study found that cortical dysplasia was observed in 27 CMV-positive patients (50.0%). Therefore, CP was found to be associated with cortical dysplasia and marked intellectual disability. Moreover, CMV infection is considered as a cause of various neurological sequelae. All these reults suggeste that brain MRI investigations and the test of congenital CMV infection are important for making a diagnosis and formulating an intellectual prognosis of children with CP [53].

In this study, CMV-IgG and IgM in the serum of mother was positive identified by TORCH test during pregnancy, CMV-IgG and IgM in the serum of children was positive identified by TORCH test, and the urinary CMV-DNA data was also collected. Previous study suggested that detection of CMV-IgG and IgM in the serum of pregnant women was necessary for the diagnosis of maternal primary cytomegalovirus infection [54]. In addition, urine is also consider to be reliable specimens for neonatal cytomegalovirus screening using PCR [55]. According these evidences, the DNA of CMV in the urine and the anti-CMV IgG and IgM in the serum of mother and children can allow the diagnosis of intrauterine infection.

Intrauterine infection is considered as a risk factor for CP. Previous study demonstrated that congenital CMV infection in the newborn period was highly prevalent among children with CP [13]. Another study showed that congenital infection was uncommon in cases of CP and controls, however, CMV was significantly associated with CP [56]. It has been suggested the viruses may have the potential to cross the placenta and blood-brain barrier, infect the fetal brain and cause damage to developing neural tissue [57]. The viruses may have the potential to cross the placenta and blood-brain barrier, infect the fetal brain and cause damage to developing neural tissue [57]. However, further studies are needed to investigate the mechanisms and contribution of congenital CMV to the causal pathways to CP.

There are several limitations in our study. Firstly, this study is a retrospective study. Further large, prospective studies are needed in various types of CP involving different ages and severities. Secondly, we found that individuals with CP had different types of MRI findings. The method of diffusion tensor images (DTI) can characterize the damages of the white matter, especially motor tracts [58]. Therefore, the pattern of white matter abnormalities in the whole brain of CP and their correlations with the clinical scale metrics are needed to be assessed. Thirdly, large, population-based studies about the relationships between intrauterine CMV infection and clinical symptoms, white matter abnormalities in children with different types of CP are needed. Finally, as most of the children included in the present paper had 1 year of age only, the motor pattern and therefore the clinical classification of CP could change over time.

## Conclusion

In conclusion, the results of our study demonstrated that individuals with CP have higher level of intrauterine CMV infection. Moreover, the severity of CMV infection is negatively correlated with the functional outcomes of children with CP. Therefore, infection is a risk factor for the occurrence of CP and intrauterine CMV infection should be taken seriously and treated. Early control of infection may reduce the disability of children with CP.

## Data Availability

The data used to support the findings of this study are available from the corresponding author upon request.

## Abbreviations

CP: cerebral palsy
CMV: Cytomegalovirus
GDS: Gesell developmental scale
MRI: Magnetic resonance imaging
HIE: Hypoxic ischemic encephalopathy
GDS: Gesell developmental scale
DQ: Developmental quotient
